# Phylogeny and Genetic Diversity of Philippine Native Pigs (*Sus scrofa*) as Revealed by Mitochondrial DNA Analysis

**DOI:** 10.1007/s10528-022-10318-0

**Published:** 2023-01-09

**Authors:** Joy B. Banayo, Kathlyn Louise V. Manese, Agapita J. Salces, Takahiro Yamagata

**Affiliations:** 1 Animal Genetics and Breeding, Graduate School of Bioagricultural Sciences, Nagoya University, Furo-Cho, Chikusa, Nagoya, 464-8601 Japan; 2 Animal Breeding Division, Institute of Animal Science, College of Agriculture and Food Science, University of the Philippines Los Baños, College, 4031 Laguna Philippines

**Keywords:** Mitochondrial DNA D-loop region, Indigenous pig, Livestock, Biodiversity, Haplotype, Conservation

## Abstract

Philippine native pigs (PhNP) are small black pigs domesticated in rural communities in the Philippines. They are valued locally for their various sociocultural roles. Recently, considerable literature has accumulated in the field of native pig production and marketing. However, there is limited research on the genetic diversity of PhNP. No previous study has investigated the evolutionary relatedness among native pigs from various islands and provinces in Luzon and the Visayas, Philippines. In addition, a much debated question is whether the PhNP were interbreeding with or even domesticated from endemic wild pigs. This study aims to clarify some of the uncertainties surrounding the identity and classification of PhNP based on mitochondrial DNA (mtDNA) signatures. Native pig samples (*n* = 157) were collected from 10 provinces in Luzon and the Visayas. Approximately 650 base pairs of the mtDNA D-loop region were sequenced and analyzed together with publicly available sequences. Pairwise-distance analysis showed genetic separation of North and South Luzon (SL) and the clustering of SL with Visayan pigs. Phylogenetic analysis showed that the PhNP clustered within 3 recognized Asian pig domestication centers: D2 (East Asia), D7 (Southeast Asia) and the Cordillera clade (sister to the Lanyu). We identified 19 haplotypes (1–38 samples each), forming 4 haplogroups, i.e., North Luzon, South Luzon and Visayas, Asian mix and the Cordillera cluster. No endemic wild pig mtDNA was detected in the native pig population, but evidence of interspecific hybridization was observed. This study showed that the Philippine native pigs have originated from at least 3 *Sus scrofa* lineage and that they were not domesticated from the endemic wild pigs of the Philippines.

**Supplementary Information:**

The online version contains supplementary material available at 10.1007/s10528-022-10318-0.

## Introduction

The Philippine native pig (PhNP) is a small black pig domesticated in rural communities in the Philippines. They were introduced around 4000 years ago (Amano et al. 2013; Piper et al. 2009b) and are currently valued for their heat tolerance, disease resistance and meat quality (Santiago et al. 2016). As such, the Congress of the Philippines has passed a local legislation (Senate Bill 821 Philippine Native Animal Development Act of 2019) that promotes their conservation and development. This bill defines the native animals as “refer[s] to breeds of chickens, pigs, cattle, goats, sheep, ducks and other domesticated farm animals that are more adapted to the environmental conditions of the Philippines, having emerged through a long process of natural selection”. Therefore, the word ‘native’ was used as early as 1980 (Eusebio 1980; Maddul 1991; Penalba and Jirel 1993; Bondoc 1998; Bondoc 2008; Basilio 2016; Dichoso et al. 2022) pertaining to the adaptability of the animals to local conditions and their important sociocultural role, rather than its evolutionary context. True native and endemic pigs in the Philippines are referred to as wild pigs, i.e*., Sus philippensis, S. cebifrons, S. oliveri, and S. ahoenobarbus* (Heaney et al. 2020; Ingicco et al. 2017; Lucchini et al. 2005; Groves 1997).

The utility of the native pig varies depending on the geographic location within the country. There are at least 7641 islands (Larena et al. 2021; NAMRIA 2017, 2018) in the Philippines and at least 100 ethnic groups (Reyes et al. 2016) and 179 dialects (PSA 2022). In the highland provinces of the Cordillera region, native pigs are considered an important sociocultural component and are used in rituals and festivities (Lapeña and Acabado 2017). Various ethnic meat products, such as *etag* and *kiniing*, have been developed from this animal. Recently, there has been renewed interest in the native pig for the *lechon* (whole-roasted pig) delicacy in the mainstream market, for which native pigs are preferred over transboundary breeds due to the superior quality of their meat. Taken together, these developments suggest that the native pig can play a crucial role in contributing to food security and livelihood in rural communities.

Previous genetic studies have shown that native pigs from Batanes (Northern Philippines) (Li et al. 2017), South Luzon (Dichoso et al. 2022) and Visayas (Layos et al. 2022a, b) possess ancient mtDNA signatures initially found in the miniature Lanyu breed of Taiwan (Wu et al. 2007a). Layos et al. (2022b) further identified an A143T mutation that distinguishes the Philippine native from the Lanyu. Microsatellite analysis showed that they are distinct from Duroc and Yorkshire but maybe related to the Berkshire (Oh et al. 2014). Furthermore, farm productivity of several native pig populations showed subtle differences (DOST 2017). The current study will compare the genetic relatedness among pigs in Luzon, Visayas, and global breeds to support local efforts on native pig conservation (Philippine Native Animal Development Act of 2019; Santiago et al. 2016; Monleon 2005). In addition, we intend to clarify the much debated question on whether native pigs were interbreeding with or even domesticated from the endemic wild pigs in the Philippines (Eusebio 1980; Bondoc 1998, 2008).

This study aims to clarify some of the uncertainties surrounding the ancestry and relatedness of native pigs in the Philippines. We will examine native pigs from 10 provinces using mtDNA signatures.

## Materials and Methods

### Samples and Sampling Site

**Fig. 1:**
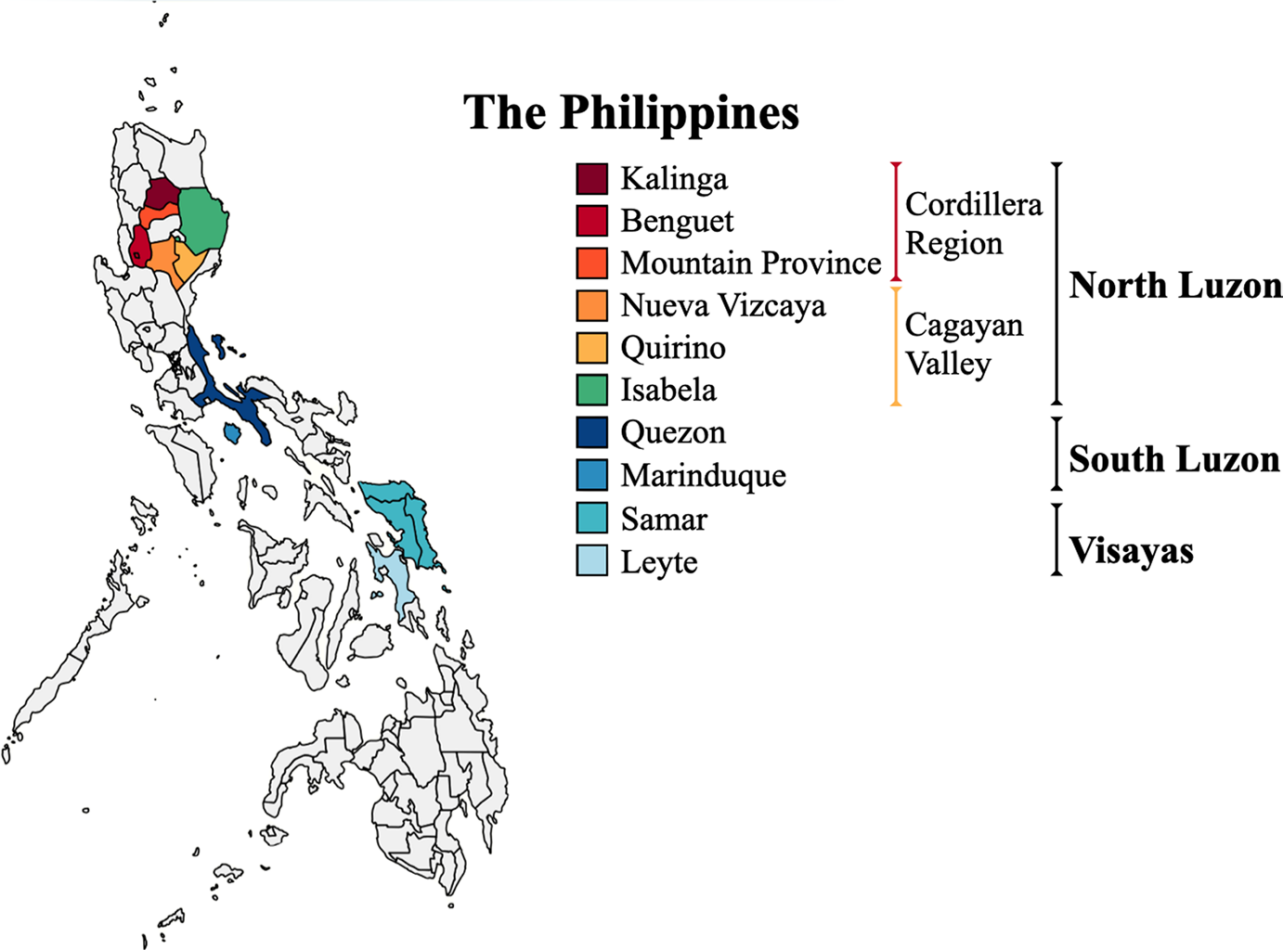
Sampling sites of the native pigs in the Philippines (Color figure online)

PhNP samples (ear notch tissue or hair follicle) were collected from 10 provinces in the Philippines (Fig. 1) following FAO (2011) guidelines on the use of unrelated individuals and unbiased sampling. Ear tissue was clipped from the ear and stored in ethanol at −20 °C until needed. This study included a total of 200 pig samples (*n* = 157 native, *n* = 43 transboundary breeds) collected from *(i)* smallholder farms in Benguet (*n* = 15), Mountain Province (*n* = 7), Kalinga, (*n* = 32), Isabela (*n* = 22), Nueva Vizcaya (*n* = 17), Quirino (*n* = 3), Quezon (*n* = 21), Marinduque (*n* = 20), combined Eastern, Northern and Western Samar (*n* = 18), Leyte (*n* = 2), *(ii)* the National Swine and Poultry Research and Development Center, Bureau of Animal Industry (BAI), Tiaong Stock Farm, Tiaong, Quezon for Berkshire (BAI BS; *n* = 10), *(iii)* BAI-accredited swine breeding farms in the Philippines for purebred BS (*n* = 3), Landrace (LR; *n* = 10), Large White (LW; *n* = 10) and Duroc (DC; *n* = 10).

### DNA Extraction and PCR Amplification

Ear notch samples (20 mg) or hair follicles (5 pieces) were rehydrated in phosphate-buffered saline and extracted using a GF-1 Tissue DNA Extraction Kit (Vivantis Technologies, Malaysia) following the manufacturer’s protocol. The partial mtDNA D-loop region (about 650 base pairs (bp)) was amplified using a single pair of primers: forward 5′-CAACCAAAACAAGCATTCCATTC-3′, reverse 5′-ATGAGTTCCATGAAGTCCAGC-3′. PCR was prepared in 25 μL reactions using 2×*Taq* Mastermix (Vivantis Technologies, Malaysia) containing a final concentration of 1 U *Taq* DNA polymerase, 1.5 mM MgCl_2_, 0.2 mM dNTPs, and 0.2 μM each of the forward and reverse primers and 20 ng DNA. The PCR products were sequenced directly by double pass standard sequencing with purification by a third-party service provider (Macrogen Inc., Seoul, South Korea). Nucleotide sequence data reported are available in the DDBJ/EMBL/GenBank databases under the accession numbers OM363266—OM363454.

### Additional Sequences

Additional sequences were downloaded from the NCBI Nucleotide and RefSeq database (NCBI Resource Coordinators 2016; O'leary et al. 2016) including the following: Panay (MN625805-28, *n* = 23, Layos et al. 2022b), Batanes (KP987306-7, *n* = 2, Li et al. 2017), *S. cebifrons* (NC_023541.1), African warthog *Phacochoerus africanus* (NC_008830.1, Wu et al. 2007b), domestic and wild pigs from Europe and Asia (Table S1).

### mtDNA Genetic Diversity (*p*-Distances)

Genetic divergence was computed within groups and between groups in MEGA X v10.2.4 using the Tamura 3-parameter model with 1000 bootstrap replicates (Kumar et al. 2018). Sequences were first grouped into 6 Philippine subpopulations (*n* = 19 to 50) and 4 transboundary breeds (DC *n* = 26, BS *n* = 17, LR *n* = 31 and LW *n* = 47). Samples were pooled to create the subpopulations Cordillera (Benguet, Mountain Province, Kalinga), Cagayan Valley (Isabela, Nueva Vizcaya and Quirino), and Samar (Eastern, Western, Northern Samar, Leyte). The rate of variation within each site was modeled with a gamma distribution (shape parameter 0.39). All ambiguous positions were removed from each sequence pair (pairwise deletion). A total of length of 577 bp were used in the final dataset.

### Phylogenetic Analysis

Sequences (*n* = 785 taxa, of which *n* = 191 are from this study after successful sequencing, Table S1) were aligned by MUSCLE in MEGA X software v10.2.4. DNA sequences were trimmed to equal length, and gaps were deleted (398 and 538 positions tested). Phylogeny was constructed by various methods, such as neighbor-joining (NJ), maximum likelihood (ML), and minimum evolution (ME), with 1000 bootstrap replicates each. The DNA substitution model of Tamura and Nei 1993 (TN93) was identified as best for the data based on the lowest BIC value in the Find Best DNA Model option in MEGA X. The substitution matrix was estimated using TN93 with assumptions of 5 gamma categories and heterogeneity patterns, where gaps were treated as complete deletions. A subset of 367 taxa was used to reconstruct a more refined tree using the Tamura 1993 model of DNA substitution with all sites used. The phylogenetic tree was rooted by *P. africanus.*

### Haplotype Analysis

Two haplotype networks were created using 672 taxa at 577 bp length and using a subset of 290 taxa at 538 bp length. Haplotype networks were constructed by median-joining using Population Analysis with Reticulate Tress (PopART) (Leigh and Bryant 2015; Bandelt et al. 1999) software. The Philippine haplotype sequence, number of haplotypes (h), number of polymorphic sites (S), haplotype diversity (Hd), and Tajima’s *D* were generated in DNA Sequence Polymorphism (DnaSP) v6.12.03 (Rozas et al. 2017) software using a length of 538 bp of 175 PhNP sequences (from this study and Genbank).

## Results

### Genetic Divergence

The genetic divergence among the pig breeds was analyzed by pairwise distances. The native pigs were found to be diverged from DC (0.0167) and LR (0.0161), but may not be diverged from LW (0.0019) and BS (0.0016) (Table 1). Among the native pigs, Quezon population was most diverged from BS (0.0059) and LW (0.0060). Native pig populations closest (below 0.0030) to the BS were Cordillera, Cagayan, and Panay, and those to LW were Marinduque and Panay. Pairwise distances among transboundary breeds were 0.0001–0.0119, and among native pigs were 0.0001–0.0079. Furthermore, among the native pigs, genetic distances of intermediate values (0.0055–0.0079) separated the North Luzon from the South Luzon—Visayas populations. Short distances (≤ 0.0005) were observed between Cordillera and Cagayan Valley (in North Luzon) and among the islands of Marinduque, Samar and Panay (in South Luzon—Visayas). Quezon pigs were relatively closer to Samar and Marinduque (0.0008–0.0012) and most distant from Panay (0.0016). On the other hand, within-group distances ranged from 0.0058 (Marinduque) to 0.0140 (Cordillera) among PhNP, and from 0.0080 (DC) to 0.0120 (BS) among transboundary breeds.

**Table 1:** Estimates of genetic divergence by *p*-distances within and between populations of Philippine native and  transboundary pigs

Populations	Sub-populations	Within group (distance)	Net between groups (distance)										
	Philippine native (*n* = 177)	0.0130 ± 0.0029	Philippine native	North Luzon		South Luzon		Visayas		Transboundary			
				Cordillera	Cagayan Valley	Quezon	Marinduque	Samar	Panay	Duroc	Berkshire	Landrace	Large White
North Luzon	Cordillera (*n* = 52)	0.0140 ± 0.0037	**–**	–									
	Cagayan Valley (*n* = 43)	0.0139 ± 0.0035	**–**	0.0003	–								
South Luzon	Quezon (*n* = 19)	0.0088 ± 0.0019	**–**	0.0070	0.0079	–							
	Marinduque (*n* = 19)	0.0058 ± 0.0023	**–**	0.0055	0.0065	0.0012	–						
Visayas	Samar (*n* = 20)	0.0068 ± 0.0024	**–**	0.0058	0.0063	0.0008	0.0005	–					
	Panay (*n* = 24)	0.0068 ± 0.0023	**–**	0.0052	0.0058	0.0016	0.0001	0.0002	–				
Transboundary	Duroc (*n* = 36)	0.0080 ± 0.0021	0.0167 ± 0.0045	0.0159	0.0153	0.0236	0.0210	0.0211	0.0203	–			
	Berkshire (*n* = 17)	0.0120 ± 0.0029	0.0016 ± 0.0005	0.0029	0.0023	0.0059	0.0035	0.0034	0.0027	0.0110	–		
	Landrace (*n* = 31)	0.0087 ± 0.0023	0.0161 ± 0.0046	0.0158	0.0152	0.0221	0.0200	0.0199	0.0194	0.0001	0.0102	–	
	Large White (*n* = 47)	0.0111 ± 0.0029	0.0019 ± 0.0005	0.0037	0.0038	0.0060	0.0024	0.0033	0.0020	0.0119	0.0007	0.0117	–
	Mean distance		0.0163 ± 0.0034										

### Phylogenetic Analysis

**Fig. 2:**
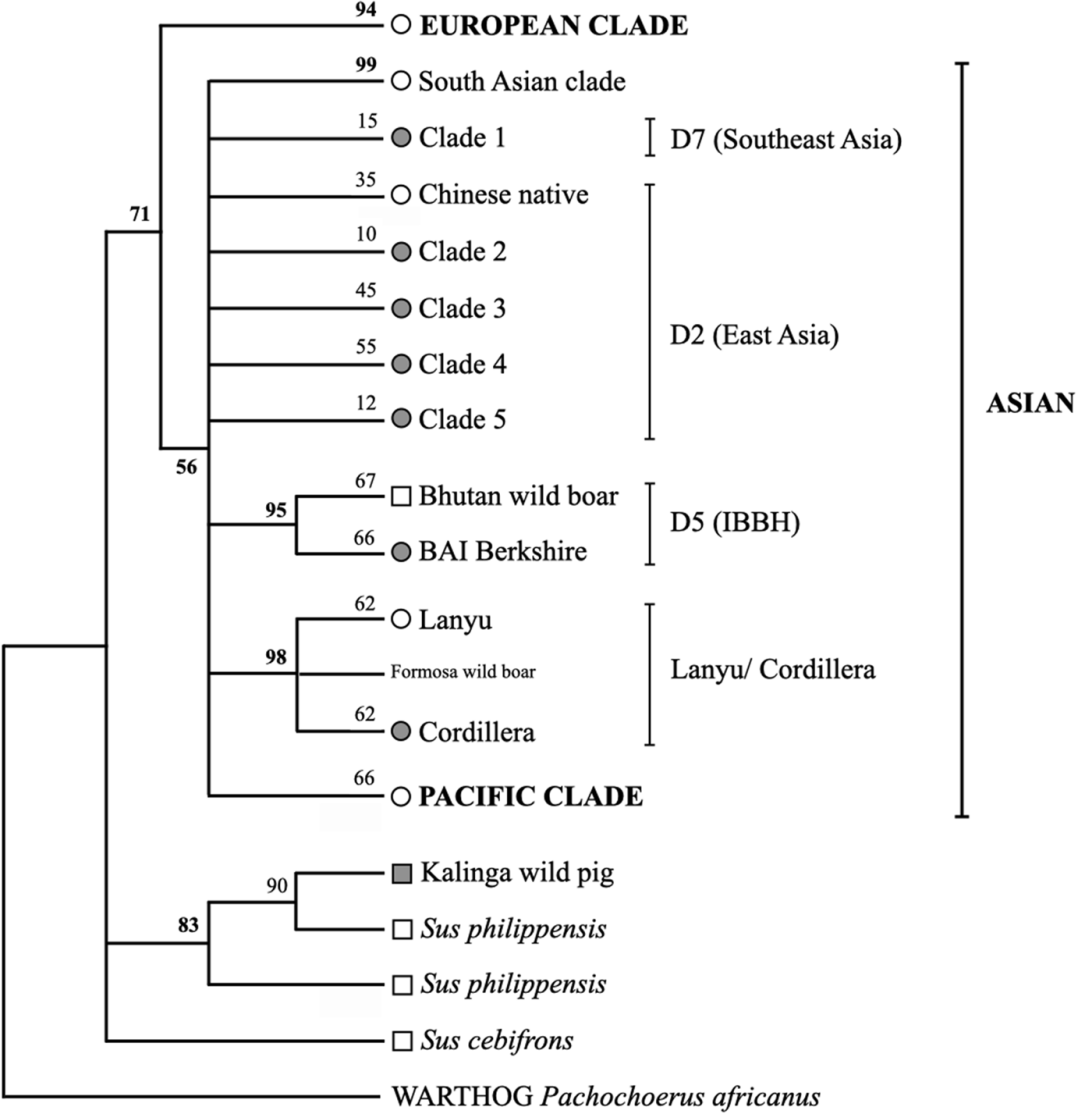
Phylogenetic tree of mtDNA signatures from Philippine native, Asian, European and wild pigs. Percentage bootstrap support is shown next to the branches. D2, D5, and D7 are indicated according to Larson et al. (2005) and Layos et al. (2022a). Node marker: circles- domestic pigs, squares- wild pigs and wild boars, gray fill- Philippine samples collected for this study

Phylogenetic analysis confirmed that the PhNP belong to the *S. scrofa* Asian clade (Fig. 2), which can be further divided into 6 subclades (i.e., Clade 1–5 and Cordillera clade). Clade 1 included pigs from Quezon, Marinduque, Samar and Nicobari Island. Clade 1 also contained PhNP of D7 haplotype previously identified by Layos et al. (2022a, b). Clade 2 included 18 pigs from various provinces in North Luzon and Asian (Formosan wild boar and native pigs from China, Vietnam, Sri Lanka and India), BS and Yorkshire (YS) pigs. Clade 3–5 was composed of native pigs from various provinces, LW/YS and BS, among others. The Cordillera clade had a distinct node (98% bootstrap) composed of 38 pigs from the Cordillera region (Kalinga, Benguet, Mountain Province), Nueva Vizcaya, Isabela and Batanes Island. We reconstructed the phylogenetic tree using a shorter sequence (398 positions) and more taxa and arrived with the same result (data not shown).

On the other hand, our DC and LR samples clustered within the European clade (D1), while LW and BS clustered within the general Asian clade (D2). However, BAI BS clustered distinctly with Bhutan wild boar, such as HQ318431 belonging to D5 (Larson et al. 2005; Nidup 2011) and was therefore excluded from further analysis. In addition, a single Kalinga sample (OM363266) clustered with the *S. philippensis* clade with 90% bootstrap support (Fig. 2) and was also excluded from further analysis.

### Haplotype Analysis

**Fig. 3:**
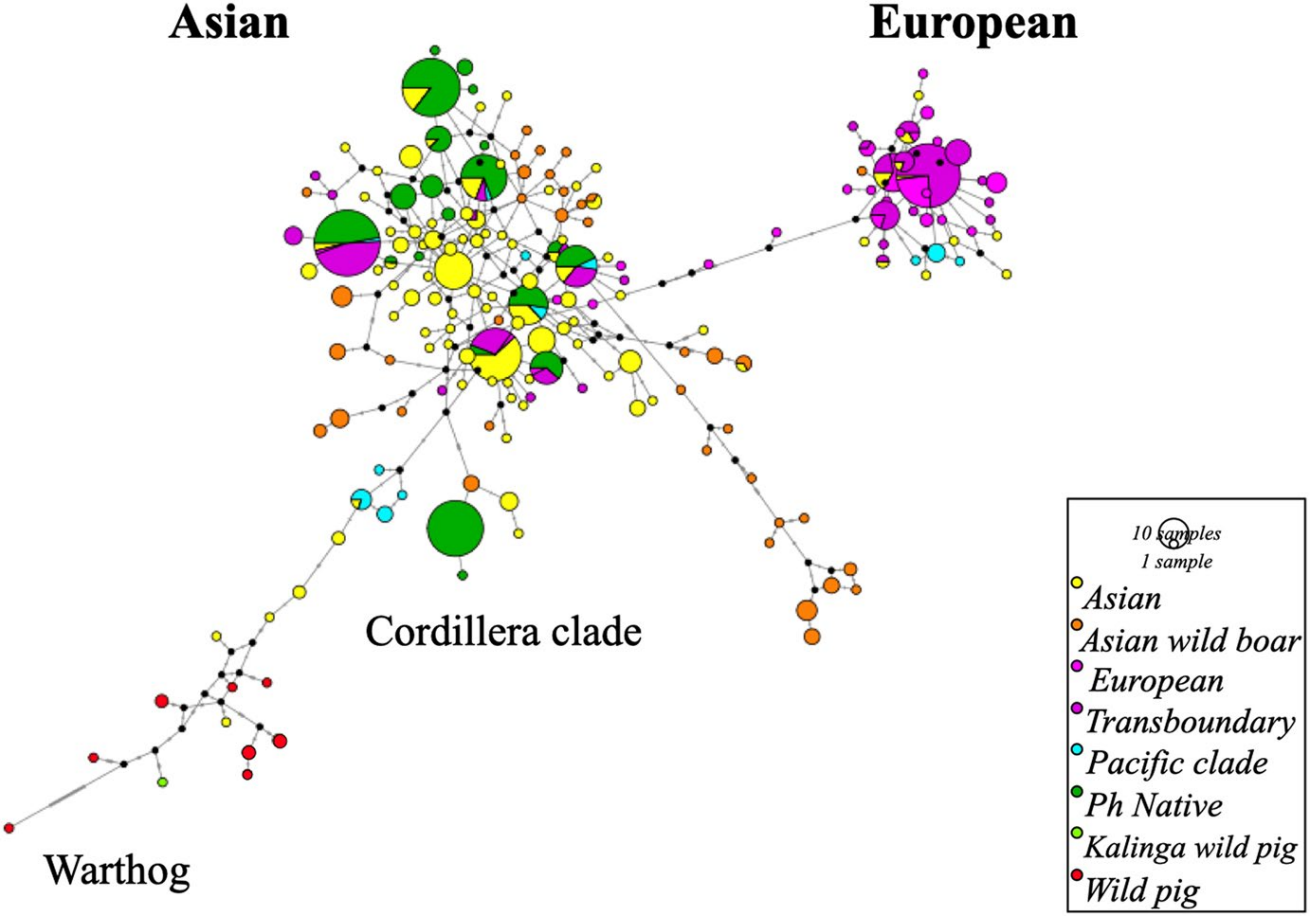
Haplotype network of mtDNA D-loop region depicting the relationship of Philippine pigs to other breeds. The Philippine (Ph) native pigs have 19 haplotypes and belong to the general Asian clade. Number of sequences used: Asian *n* = 208, Asian wild boar *n* = 69, European *n* = 54, Transboundary *n* = 134, Pacific clade *n* = 22, Ph Native *n* = 178, Kalinga wild pig *n* = 1, Wild pig *n* = 6, total *n* = 672 (Color figure online)

**Fig. 4:**
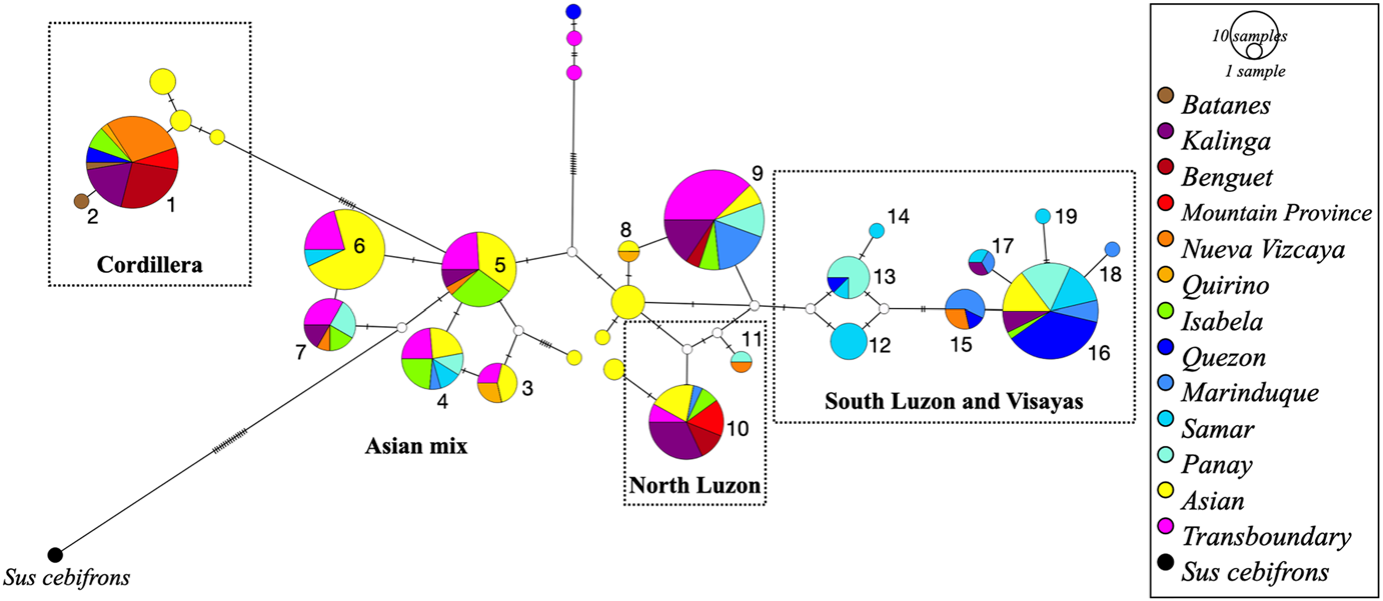
Haplotype network of mtDNA D-loop region that is either unique or shared among Philippine native, Asian and transboundary pigs. Numbers indicate Philippine (Ph) haplotypes, which can be assigned to 4 haplogroups**:** (1) Cordillera, (2) North Luzon, (3) South Luzon and Visayas, and (4) all remaining, to the general Asian mix clade. Samples used: Ph *n* = 180, Asian *n* = 67, transboundary breeds *n* = 43. Hatch marks denote single mutations. White circles are median vectors showing consensus sequences between haplotypes representing potentially unsampled or extinct relatives (Color figure online)

**Fig. 5:**
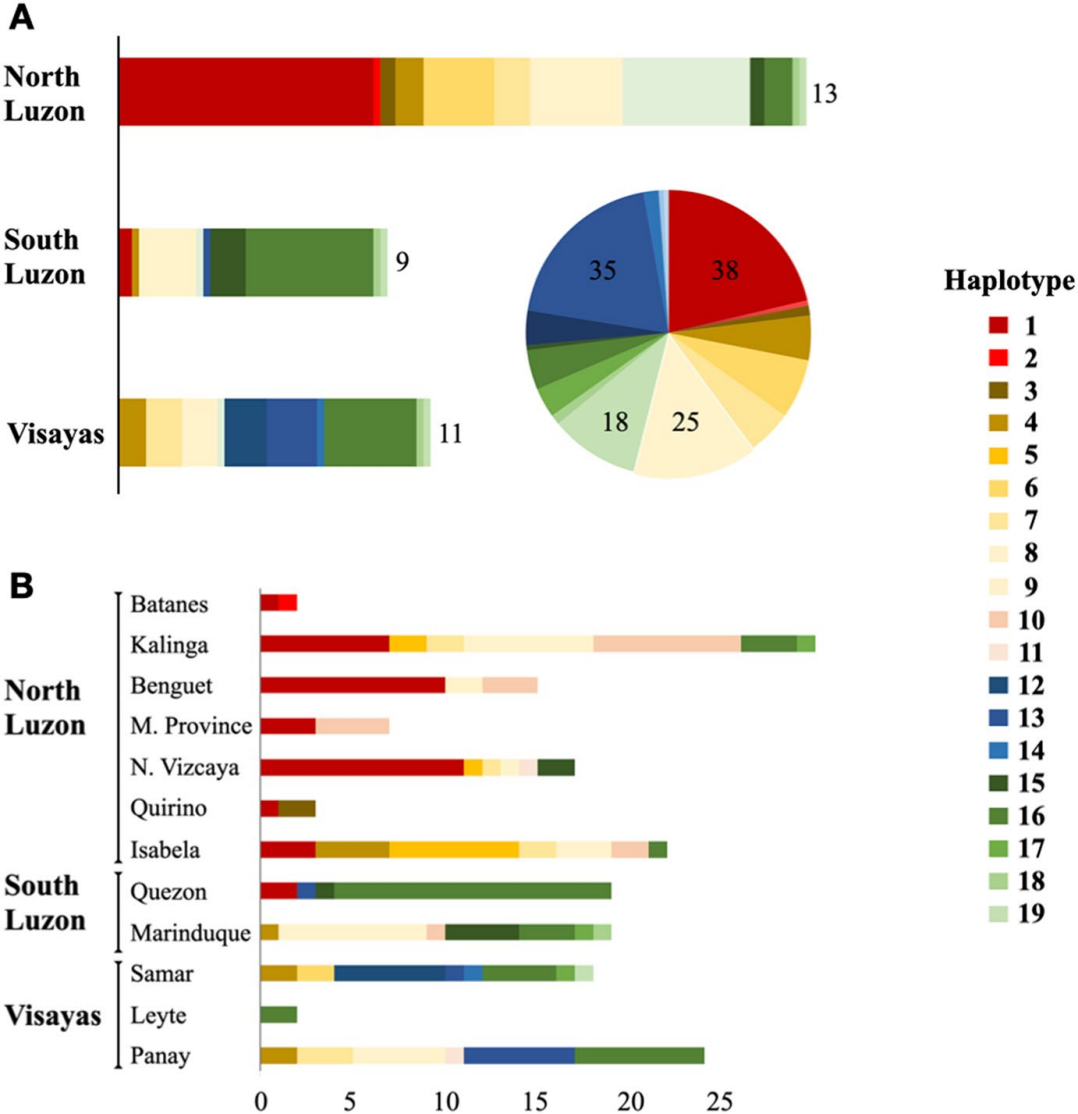
Distribution of the 19 haplotypes per Philippine region (**A**) and province (**B**). The size of the bar and pie graph is proportional to the sampling size, the number of haplotypes for each region is shown on the side of the bar graph and the sample size of major haplotypes is shown in the pie chart (**A**). M. Province - Mountain Province, N. Vizcaya - Nueva Vizcaya (Color figure online)

Haplotype analysis of the PhNP revealed 19 haplotypes, Ph_1 to Ph_19 (Figs. 3 and 4). Ten haplotypes were unique to the Philippines and nine were shared with native and wild boars from Asia (Chinese, Jeju, Lanyu, Okinawa, Nicobari, Vietnamese, Ryukyu, Celebes wild pig), Europe (Tamsworth, French wild boar, prehistoric Austrian) and transboundary breeds (LW, DC, BS). The haplotype network revealed at least 4 haplogroups, hereby referred to as the Cordillera Cluster (CC), North Luzon Cluster (NLC), South Luzon-Visayas Cluster (SLVC) and Asian Mix Cluster (AMC) (Fig. 4). The number of haplotypes per clade was 2, 2, 8, and 7 for the CC (Ph_1-2), NLC (Ph_10-11 and 2 median vectors), SLVC (Ph_12-19 and 2 median vectors), and AMC (Ph_3-9), respectively. Each haplotype was represented by 1 (Ph_14) to 38 (Ph_1) samples. Ph_9 is shared between a large proportion of LW/YS and native pigs from various provinces. The DC, LR and LW samples in this study showed European haplotypes, while the BS showed an Asian haplotype (not shown). The haplotype distribution per region was 13, 9, and 11 in North Luzon, South Luzon and the Visayas, respectively (Fig. 5A). Each province had 1 (Leyte) to 8 (Samar) haplotypes (Fig. 5B). The details of the haplotype are presented in Table S2 to S4.

## Discussion

The PhNP showed a considerable genetic distance from DC and LR but were closer to LW and BS (Table 1). Microsatellite analysis by Oh et al. (2014) showed similar results. It must be noted that LW and BS are known to have Asian mtDNA due to their intensive upgrading with Asian pigs (Giuffra et al. 2000). The genetic distances between native pig populations correlated with their geographic distance, suggesting some isolation between pigs from North Luzon and those in South Luzon and the Visayas (Fig. 1). The same trend of NL vs. SL separation was also observed among Philippine native cattle using BoLA gene markers (Takeshima et al. 2014). The higher mtDNA diversity observed in the Cordillera and Cagayan Valley (encompassing 4 Provinces in North Luzon) was due to the presence of multiple haplotypes (*n* = 13) from the 2 varying lineages (Asian and Cordillera clade) (Fig. 2).

There are at least 7 recognized pig domestication centers, denoted as D1–D7, plus other cryptic domestication sites (Larson et al. 2005; Tanaka et al. 2008; Layos et al. 2022a). Phylogenetic analysis showed that PhNP clustered to various lineages such as the ancient Cordillera/Lanyu clade and the general Asian Clade (D2 and D7) (Fig. 2). Pigs from NL clustered either in the Cordillera clade or in the D2 (East Asian) clade, while pigs from SLV clustered within the D7 (Southeast Asian). This study provides additional evidence on the multiple ancestry of the PhNP as observed by previous studies (Layos et al. 2022a, b; Dichoso et al. 2022).

Of the 19 haplotypes, the Ph_1 haplotype (CC) was the major haplotype in this study (Fig. 4). The median-joining network showed that the Ph_1 haplotype was 10 mutations and 2 median vectors away from the nearest Ph haplotype (Ph_5). It was, however, only one mutation away from the Formosan wild boar with-Lanyu-sign-lineage (KP987300). Similarly, the type I Lanyu (EF375877.3) is also characterized by a single mutation from the Formosan wild boar but at another position (Fig. 3 and 4). Layos identified a single mutation (A143T transversion) that distinguishes the PhNP from the Lanyu (Layos et al 2022a). Our data suggest that the Cordillera clade is sister to the Lanyu and hint at the potential domestication of a common ancestral wild boar in the Philippines, whether it is the Formosan or another yet undiscovered wild boar. There were speculations of an ancient land connection between [Mountain Province] Cordillera and Formosa during early Tertiary which facilitated the dispersal of several Himalayan plants into both areas at the same time and through the same channels (Merrill 1926). Central Cordillera region is believed to have originated as a series of small islands about 30 million years ago (MYA) that formed what is now the Central Cordillera (Heaney et al. 2016). Therefore, the geological history of the Cordillera region is too old to discount the natural dispersal of the pig belonging to the Cordillera and the Lanyu clade. Currently, the Cordillera is one of the most diverse and important biotas in the Philippines with many endemic genera (Heaney et al. 2016; Heaney et al. 2005). The Lanyu was estimated to have diverged at 0.6 MYA earlier than other East Asian wild boars (Li et al. 2017). The CC haplotype was the major mtDNA signature of PhNP in Benguet, Mountain Province, Kalinga and Nueva Vizcaya. These provinces are connected by the Cordillera and Caraballo Mountain Range, where indigenous communities prefer this type of pig. The CC haplotype continues into Isabela, the Palawan region and Bohol (Layos et al. 2022b), possibly by human-mediated translocation. Ancient mtDNA haplotypes were also found in Philippine native chickens (Thomson et al. 2014). We show long-term genetic continuity between early and modern domestic pigs as was observed in China (Larson et al. 2010).

In the Philippines, crosses of LW, LR, and DC were common in smallholder production systems (Peñalba 2005). These are still the main breeds used locally (DOST 2018). The shared haplotype (Ph_9) of PhNP and LW/YS may indicate localized crossbreeding for the production of *lechon* pig. Crossbreeding with transboundary breeds have replaced the mtDNA signatures of several Asian native pigs (Charoensook et al. 2019; Zhang et al. 2018, 2016). On the other hand, the shared haplotype between native and BS could be due to their shared mtDNA ancestry, since the BS possess Asian type mtDNA (Giuffra et al. 2000). It is interesting that the BS is most distant from Quezon native indicating that the *Berkjala* crossbreed was unable to contribute BS mtDNA into the modern pig population of South Luzon. The *Berkjala* was developed in the early 1900s using BS and Laguna native pig (Matias 1995; Eusebio 1980) but is presumably extinct (Arboleda et al. 2003).

Being traditionally free-ranged, the PhNP were hypothesized to be interbreeding with or even domesticated from Philippine endemic wild pigs (Eusebio 1980; Bondoc 1998, 2008). We confirm only one pig, described as wild by the owner, to be an *S. philippensis* type (Fig. 2). No wild pig mtDNA was detected in pigs described as native, corroborating the *S. scrofa* ancestry of the PhNP. Since mtDNA is only maternally inherited, the depth of hybridizations and genetic introgressions between native and endemic wild cannot be ascertained in this study. Even the wild boar mtDNA were often not detectable in modern domestic pigs (Tanaka et al. 2008; Larson et al. 2005; Cho et al. 2009; Watanobe et al. 1999). These cases prove the limitation of the mtDNA in detecting hybridizations (Sasaki and Sato 2021; Moore 1995). Furthermore, paternal lineage by Y-chromosome analysis did not show clear correlation with mtDNA clades (Choi et al. 2020). Although, gene flow between domestic *S. scrofa* breeds was higher than between domestic and wild relatives (Frantz et al. 2013), in the Philippines, however, interspecific hybridization is a major threat to the genetic integrity of the wild pig populations (Heaney et al. 2020; Meijard and Melleti 2018; Melleti et al. 2018; Tabaranza et al. 2018).

We provide evidence of the Philippine farmer’s preference for interspecific hybridizations. This close cultural relationship of the Filipino people with both wild and native pigs alike have occurred since ancient times (Piper et al. 2009a). The Philippine native cattle also have interspecific origins (Aquino et al. 2006). Interspecific and intergeneric admixtures are important biological driving forces for adaptation (Liu et al. 2019; Ai et al. 2015), thereby being favorable for native animals. The adaptability of the PhNP in low input systems could be influenced by interspecific hybridization with endemic wild pigs. Furthermore, we observed that farms intentionally keep warty pigs to mate with PhNP suggesting that interspecific hybrids are advantaged in low input farming. Thus, this poses a challenge on the co-management of native and wild pigs to address both the need for adaptability in the former and for genetic integrity in the latter. Our data provide additional evidence on the expanse and hybridizations of *S. philippensis* in Kalinga province, Philippines (Meijard and Melleti 2018; Heaney et al 2005).

## Conclusion

This study set out to examine the genetic relatedness of the PhNP to clarify their identity and classification. The mtDNA of PhNP did not show close affinity to that of the endemic wild pigs of the Philippines, indicating that the PhNP had other genetic origins. Further analysis revealed its multiple ancestral origins, such as from East and Southeast Asia. We observed local preference for interspecific hybridizations potentially to improve the adaptability of PhNP. This study contributes to our understanding of Asian domestic pig ancestry, hybridization, and dispersal.

## Supplementary Information

Below is the link to the electronic supplementary material.

Supplementary file1 (DOCX 101 KB)

## Data Availability

Nucleotide sequence data reported are available in the DDBJ/EMBL/GenBank databases under the accession numbers OM363266—OM363454. All data will be available on reasonable request.
